# Inhabited subsurface wet smectites in the hyperarid core of the Atacama Desert as an analog for the search for life on Mars

**DOI:** 10.1038/s41598-020-76302-z

**Published:** 2020-11-05

**Authors:** Armando Azua-Bustos, Alberto G. Fairén, Carlos González Silva, Daniel Carrizo, Miguel Ángel Fernández-Martínez, Cristián Arenas-Fajardo, Maite Fernández-Sampedro, Carolina Gil-Lozano, Laura Sánchez-García, Carmen Ascaso, Jacek Wierzchos, Elizabeth B. Rampe

**Affiliations:** 1 Centro de Astrobiología (CSIC-INTA), 28850 Madrid, Spain; 2 Instituto de Ciencias Biomédicas, Facultad de Ciencias de la Salud, Universidad Autónoma de Chile, Santiago, Chile; 3 Department of Astronomy, Cornell University, Ithaca, NY 14853 USA; 4 Facultad de Ciencias, Universidad de Tarapacá, Arica, Chile; 5 Department of Natural Resource Sciences, McGill University, Quebec, Canada; 6 Atacama Research, Santiago, Chile; 7 Laboratory of Planetology and Geodynamics, Université de Nantes, 44322 Nantes, France; 8 Museo Nacional de Ciencias Naturales (CSIC), 28006 Madrid, Spain; 9 Astromaterials Research and Exploration Science Division, NASA Johnson Space Center, Houston, TX USA

**Keywords:** Environmental microbiology, Astrobiology

## Abstract

The modern Martian surface is unlikely to be habitable due to its extreme aridity among other environmental factors. This is the reason why the hyperarid core of the Atacama Desert has been studied as an analog for the habitability of Mars for more than 50 years. Here we report a layer enriched in smectites located just 30 cm below the surface of the hyperarid core of the Atacama. We discovered the clay-rich layer to be wet (a phenomenon never observed before in this region), keeping a high and constant relative humidity of 78% (a_w_ 0.780), and completely isolated from the changing and extremely dry subaerial conditions characteristic of the Atacama. The smectite-rich layer is inhabited by at least 30 halophilic species of metabolically active bacteria and archaea, unveiling a previously unreported habitat for microbial life under the surface of the driest place on Earth. The discovery of a diverse microbial community in smectite-rich subsurface layers in the hyperarid core of the Atacama, and the collection of biosignatures we have identified within the clays, suggest that similar shallow clay deposits on Mars may contain biosignatures easily reachable by current rovers and landers.

## Introduction

Three rovers will land on Mars in the next years (Perseverance, Rosalind Franklin and Tianwen-1) with two of them (Perseverance and Rosalind Franklin) having the primary goal of seeking preserved biosignatures in clays^1^. NASA’s Mars2020 *Perseverance* rover will land in Jezero crater^2^, an impact crater with a diameter of about 45 km located at the edge of the Isidis Basin on Mars. Orbital images suggest Jezero was the site of an open-basin lake ~ 4 Gyr ago during the Noachian era^3–5^. The Compact Reconnaissance Imaging Spectrometer for Mars (CRISM) onboard NASA’s Mars Reconnaissance Orbiter identified iron/magnesium-smectites and magnesium-rich carbonates within the Jezero crater fan deposits^6,7^ thought to have been transported from the surrounding Nili Fossae region^8,9^ (smectites are a well-known group of phyllosilicates, or clay minerals, part of a class of “swelling clays” that can readily exchange H2O and cations in their structures^10^). ESA’s *Rosalind Franklin* rover will land on Oxia Planum in 2023^11^, a Noachian terrain located on the southwest margin of Arabia Terra^12^. The smectite-rich unit at Oxia is representative of a more widespread aqueously altered unit, exposures of which are found scattered over distances as large as 1900 km and which include the Mawrth Vallis clay-rich region^13^. In addition, NASA’s *Curiosity* rover, which landed in Gale crater in August 2012, has already investigated an Fe/Mg smectite-bearing unit that was first identified from orbit^14,15^.

The reason for sending rovers to smectite-bearing Martian terrains is two-fold: (1) smectite forms from water–rock interactions, and liquid water is a prerequisite for life as we know it on Earth^16,17^, and (2) swelling clays may enable enhanced preservation of biosignatures^18^. On Mars, phyllosilicates are important secondary minerals in ancient terrains^19^, with orbital and *in-situ* data indicating phyllosilicate-bearing deposits (e.g., Jezero crater, Oxia Planum, and portions of Gale crater), suggesting that past aqueous environments preserved by these deposits had a relatively high water activity and may have been habitable to microbial life^11,16,20^. Studies of organics in smectite-bearing soils and shales on Earth demonstrate smectite can preserve organic molecules because of their reactive surfaces and ability to incorporate polar and non-polar organic molecules into the interlayer site^21–23^. Organic molecules have been identified in situ in ~ 3.5 Gyr old smectite-bearing mudstone in Gale crater, but it is not known if and how smectite played a role in their preservation^24,25^. Furthermore, although the surface of Mars today is unlikely habitable, the astrobiology community will be able to inspect for the first time the habitability of subsurface smectites in depth with the ExoMars Drill Unit^26^.

**Figure 1:**
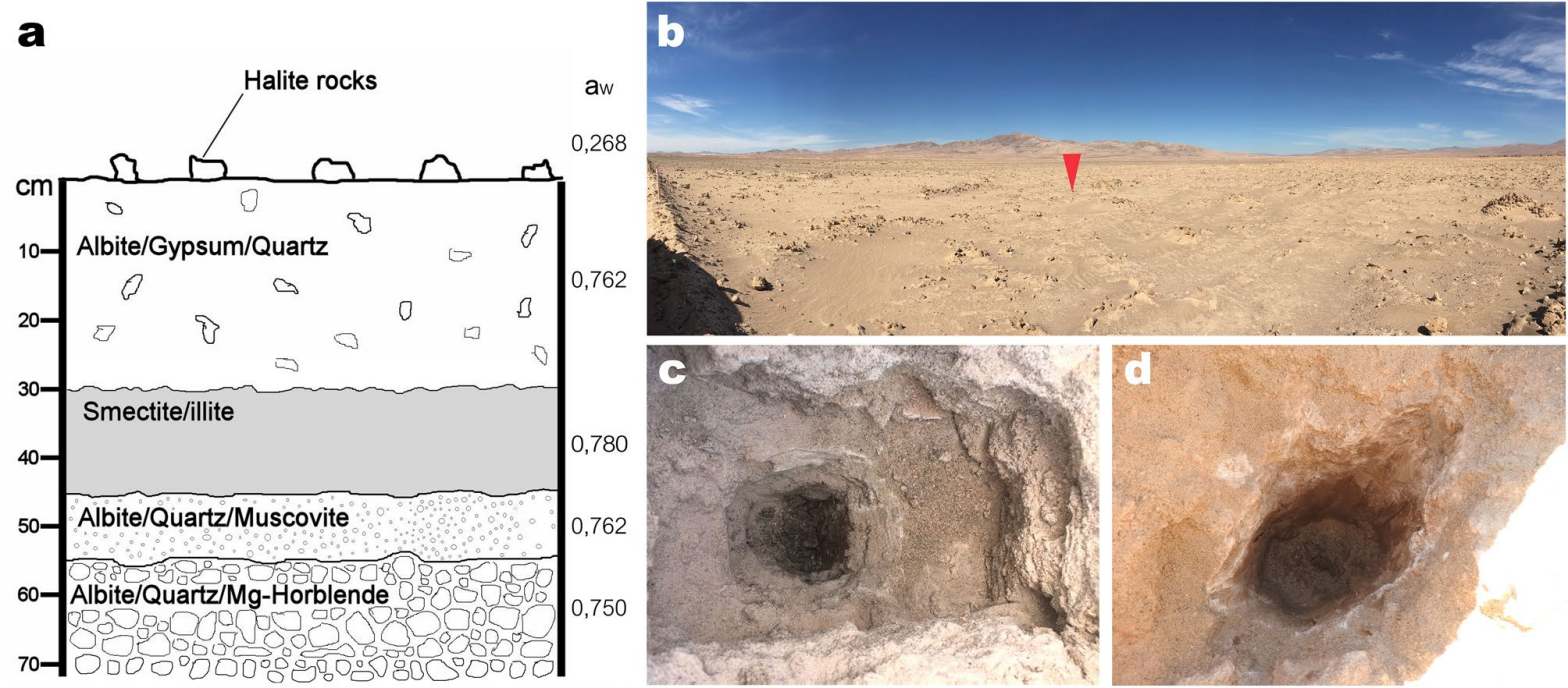
Characteristics of the study site. (**a**) Diagram of the structure, composition and mean water activity of soil layers with depth. (**b**) General view of the sampled site. The red arrow marks the first pit from where samples were taken, seen in detail in (**c**). (**d**) Another pit dug 4 km away from the pit shown in (**c**). Pit diameters are approximately 1 m length, 60 cm wide and 60 cm deep. Both (**c**, **d**) show a clear color change between the lower wet clay-rich layer and the upper soil layers.

The Atacama Desert, particularly the Yungay region, is a well-known Martian analog because of its hyper-aridity and similar salt and phyllosilicate contents^,^^27–29^, and previous studies have preliminary identified smectite in Yungay subsurface soils^29–31^. These studies described the organic matter in smectite-rich soil horizons 10 s of cm below the surface as “fossilized” and “radiocarbon dead” and suggested the organic matter had been preserved since the time of deposition up to 2 million years ago. Although drier sites are found elsewhere in the Atacama^32^, Yungay is still considered one of the driest, most highly UV-irradiated environments on Earth, with highly oxidizing soils, extremely low organic content and extremely low populations of microbial life (10^1^–10^3^ colony forming units (CFU) per gram of soil)^27,33–35^. Thus, given the critical role of smectites for preserving evidences of life, we analyzed subsurface smectite-rich soil horizons in pits dug in the hyperarid Yungay region (Fig. 1) in order to characterize its environmental conditions, assess its habitability and its potential for preserving biosignatures.

## Results and discussions

Our first observation in the studied area took place in the second week of March 2017, while sampling soils in a pit dug in the middle of Yungay (24º5′5.28″S, 69º54′54.25″W) (Fig. 1a,b). Starting 30 cm below the thoroughly characterized halite, gypsum, albite and quartz-rich soil surface and shallow subsurface^36–38^, we observed a distinct layer of wet clay mineral-rich soil (Fig. 1c). These wet sediments were also observed in the same pit later in March, July and August 2018. Four additional pits dug in August 2018 in a 4 km radius around the first pit also unveiled wet clay-rich soils in the subsurface (Fig. 1d), confirming the existence of a widespread and sustained phenomenon of sub-surface water availability at Yungay. To the best of our knowledge, this represents the first detection of wet subsurface clay-rich layers (or any other type of subsurface wet minerals) at Yungay or other sites of the hyperarid core of the Atacama.

**Figure 2:**
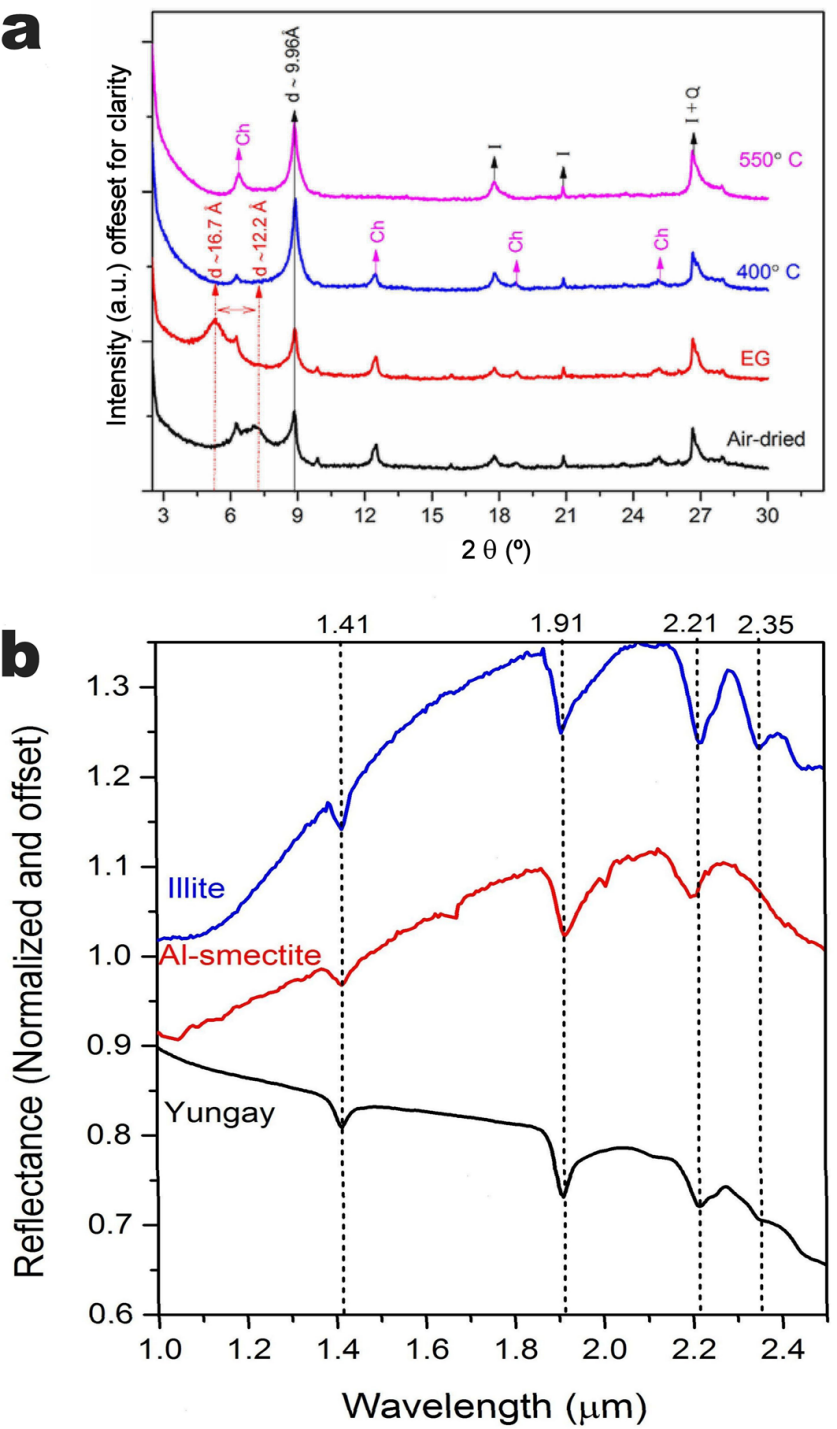
Mineralogical characterization of Yungay clay-size separates. (**a**) From bottom to top, XRD patterns of an oriented clay mount following a step-procedure to identify clay minerals: air-dried, glycolated and heated at 400 and 500 °C. The presence of a broad peak at 12.2 Å which migrated at 16.7 Å in the glycolated pattern and the presence of reflections assigned to illite (I) point to an interstratified illite–smectite specimen. The perpetuation of a sharp peak at 10 Å in all patterns indicates discrete illite is present. The sample also contains chlorite and quartz (Ch and Q). (**b**) Near-infrared spectra of Yungay clay-size separates. From bottom to top, near-infrared spectra of Yungay clay-size separate (“Yungay”), Al-smectite (montmorillonite, from CRISM spectra library) and illite (from PDS Geoscience spectra library). The Yungay clay spectrum shown is an average of three independent measurements (64 scans, 2 cm^−1^ of resolution), normalized by the maximum intensity. Dotted lines mark the vibrational bands of the clay minerals: 1.41 and 1.91 μm assigned to the combinations and overtone vibrations in OH and H_2_O; vibrational bands between 2.1 and 2.5 μm result from the distribution of cations in octahedral sites, and the bands at 2.21 μm and 2.35 μm are consistent with Al–OH and Fe–OH, respectively.

Standard gravimetric analyses of the wet clay-bearing layer confirmed they comprise 42.8 wt% consist of clay size particles. Both X-ray diffraction (XRD) of oriented clay-size (< 2 μm) aggregates and near-infrared spectra of these same samples unveiled interstratified illite–smectite as the main clay component (Fig. 2). XRD of the clay-size fraction also demonstrated the presence of discrete illite and chlorite. Samples collected from the wet clay-bearing layers also contain halite (NaCl), gypsum (CaSO_4_·2H_2_O), calcium carbonate (calcite, CaCO_3_), albite (Na(AlSi_3_O_8_)) and quartz (SiO_2_) (Supplemental Figure S1), all minerals typical of surface soils in this area^39–41^.

**Figure 3:**
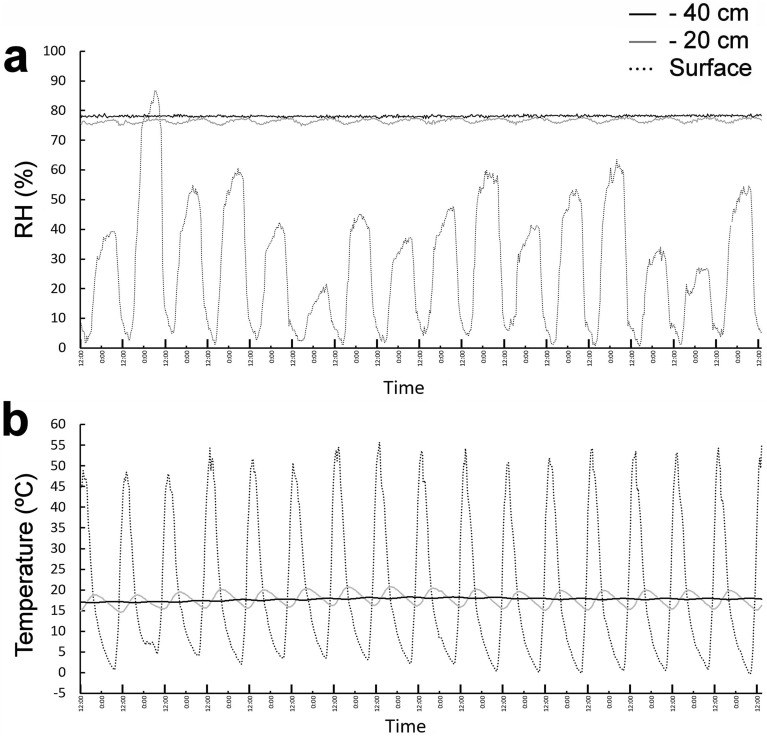
Temporal behavior of relative humidity and temperature in Yungay soil profile. (**a**) Relative humidity changes over the course of 16 days. (**b**) Temperature changes over the same time period as (**a**). Larger marks in the x axis show noon time of each day.

Dual temperature/relative humidity loggers buried in the soil profile (at the surface, 20 cm and 40 cm below the surface, this last sensor set in the middle of the clay layer) in the first inspected pit in 2018 and 2019 showed that while atmospheric relative humidity widely fluctuated daily (as it usually does in the hyperarid core of the Atacama)^32,42^, relative humidity deeper in the soil profile always remained high and constant over time (Fig. 3a). The sensor set 20 cm below the soil surface showed some variations in relative humidity (Fig. 3a), reflecting a small influence of atmospheric changes in the soil at this depth. However, the sensor set in the clay-rich layer, 40 cm beneath the soil surface, showed a high relative humidity of 78.0% (a_w_ 0.780), which remained constant for the measured time periods (see Methods). This value is much higher than the highest a_w_ values reported in Yungay surface soils, which typically vary between 0.01 and 0.524, and well above the lowest water activity limit for microbial life (a_w_ 0.6)^32^. The temperature profile over time showed that the clay-rich layer is also thermally independent of the surface atmospheric conditions (Fig. 3b); temperatures on the soil surface varied amply between 0 and 54 °C depending on the time of day, while the sensor at a depth of 20 cm fluctuated between 14 and 19 °C. In contrast, the sensor set in the clay-rich layer at a depth of 40 cm maintained a constant temperature around 17 °C, with only minor variations over time (± 1 °C). A statistical analysis of the influence of atmospheric surface temperatures on surface relative humidity in the soil profile confirmed our observations: temperature and relative humidity at the surface show a strong inverse correlation (Supplemental Figure S2, panel a). Relative humidity at a depth of 20 cm showed a minor negative correlation with atmospheric surface temperatures (Supplemental Figure S2, panel b), while within the clay-rich layer at a depth of 40 cm, relative humidity was almost completely uncoupled from the harsh and changing surface conditions (Supplemental Figure S2, panel c).

Below the clay-rich layer, we found another layer composed of granular material (a_w_ 0.762), mainly containing albite (calcian), quartz and orthoclase; and, below this, a hard, rocky stratum (a_w_ 0.750) composed of albite (calcian), quartz and ferrous magnesiohornblende (Fig. 1a). The minor decrease in water activity of the layers below the clay-rich layer shows that the illite–smectite in the clay-rich layer preferentially holds water, but may also provide a source of humidity for layers below it. Standard gravimetric analyses^43,44^ of the illite–smectite-bearing samples showed that these clays hold up to 20% of their weight in water available for microbial life. The source of water for the wet subsurface clay-rich layer is likely related to unusually intense rain events in Yungay over the past decade, particularly in March 2015 when the region received ~ 35 mm of precipitation over the course of several days^45^. Later rain events even created small lagoons in Yungay in 2017, a phenomenon never observed before in this region in the past 500 years^46^.

Although the exact environmental conditions on early Mars are largely unknown, similarities in mineralogy between soils at Yungay and Noachian- and Hesperian-aged Martian terrains indicate similar conditions to those in Yungay soils may have existed on early Mars. Sulfates and chlorides have been detected from orbit and in situ by rovers and are generally indicative of arid and evaporative environments^47,48^. Many of these salts are located in depressions or impact craters and can be associated with clay minerals^49,50^. Saline waters may have been sourced from groundwater upwelling and/or surface runoff, but the identification of river and delta deposits in some of these craters suggests surface runoff was a factor in these locations, and lake level sequences in Gale crater indicate multiple drying and rewetting cycles^51^. Arid, evaporative environments, indicated by sulfates and chlorides, and local flooding from surface runoff, perhaps from melting snow and ice in the case of early Mars, followed by periods of aridity likely existed on early Mars as they do now at Yungay.

Clay-rich layer samples showed a total organic content of 0.10% dry weight (dw) (a value ten times higher than the highest organic surface carbon values measured in Yungay)^44,45^, and total nitrogen of 0.01% dw, resulting in a C/N ratio of 10, a value in the higher limit range of other reports of desert microbial biomass^46^. Stable isotopic ratios were − 23.4 ‰ (δ^13^C) and 2.3 ‰ (δ^15^N). Depleted bulk δ^13^C ratios suggest that primary carbon fixation in these wet clay-rich layers may be explained either by a subsurface acetyl-CoA pathway (bacteria and/or archaea) and/or by the downward transport of Calvin cycle products created at the surface, as endolithic metabolically active cyanobacteria have been reported inside halites of the surface of the sampled area^38,40,52^.

**Figure 4:**
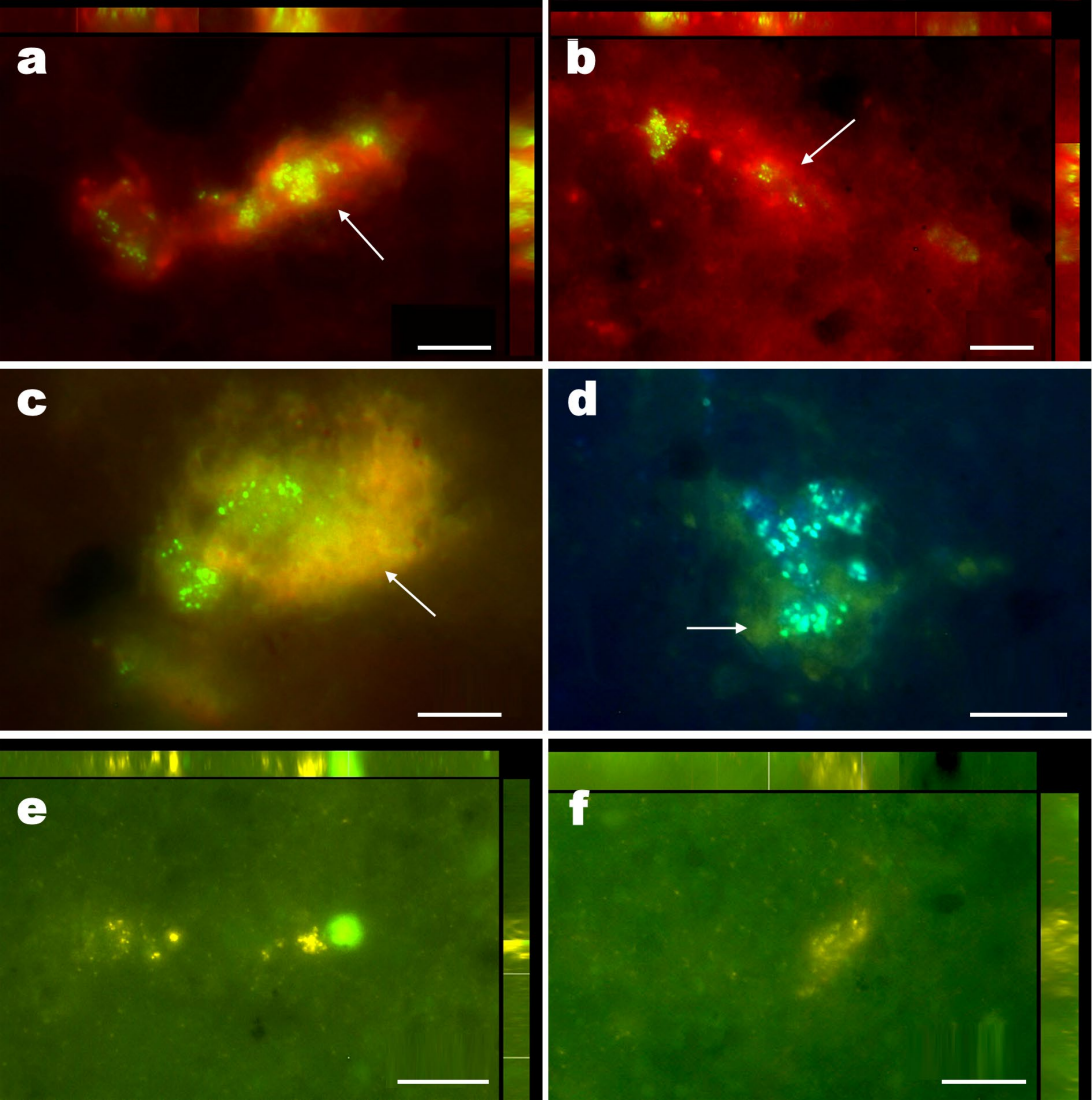
Fluorescence microscopy images of bacterial aggregates and EPS from Yungay subsurface clay samples. (**a**, **b**), Maximum image projection 3D images (upper and right bars: X and Y view of stacked sections respectively, 10 sections; z = 0.35 μm) of SYBR green stained bacterial cells (green-yellow signal) and autofluorescence red signal (arrow), potentially proceeding from exopolysaccharides. The sGFP and Rhodamine filters were used for both images (Multichannel Image Acquisition system). (**c**) Single section image of SYBR green stained bacterial cells (green signal) and autofluorescence orange (arrow) signal potentially proceeded from EPS; sGFP filter. (**d**) Single section image of SYBR green stained bacterial cells (blue signal) and autofluorescence green (arrow) signal potentially proceeded from EPS; DAPI filter. (**e**, **f**), Maximum Image Projection 3D images (upper and right bars: X and Y view of stacked sections respectively, 10 and 15 sections respectively; z = 0.35 μm) of CTC stained and metabolically active bacterial cells (yellow-orange CTF signal).

Although the heterogeneous distribution of cells in small colonies in the clay samples analyzed complicate the assessment of microbial colonization in this new habitat, we have estimated population levels of at least 9.3 × 10^3^ cells per gram of sample using direct counting after SYBR Green staining. Fluorescence microscopy analysis of clay-rich samples from 40 cm depth stained with SYBR Green also revealed that microbial cells in these clay minerals form discrete, isolated colonies encapsulated by an envelope of exopolysaccharides similar to biofilms (Fig. 4a–d), suggesting a functional collaboration among these cells^53^. In addition, CTC (5-Cyano-2,3-di-p-toly tetrazolium chloride, a redox dye commonly used as cellular indicator of respiratory activity) staining of these same samples clearly showed that these cells are actively respiring, demonstrating that these colonies are metabolically active (Fig. 4e,f), and not in a state of metabolic stasis, as it has been proposed in general for microbial species found in subsurface soils of the hyperarid core of the Atacama^54^.

**Figure 5:**
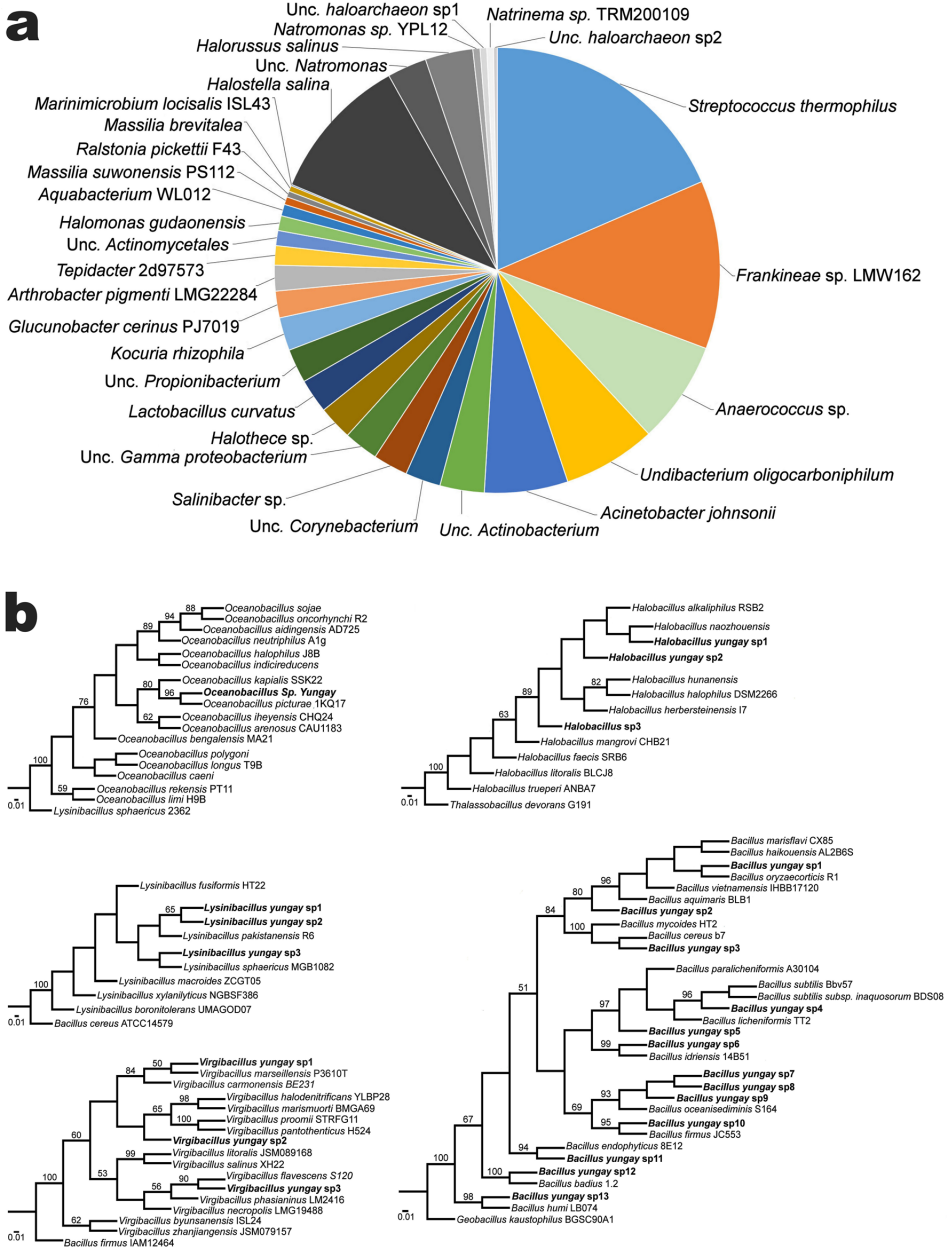
Closest phylogenetic matches of the microbial species identified in Yungay subsurface clay minerals. (**a**) Main OTUs identified from DNA extracted from Yungay subsurface clay samples. Wedges in gray denote archaeal species. (**b**) Phylip neighbor joining phylogenetic trees obtained from the aligned 16S rRNA gene sequences of clay microbial isolates (*Oceanobacillus*, *Halobacillus*, *Lysinibacillus*, *Bacillus* and *Virgibacillus*) and related species. Numbers on the nodes represent bootstrap values with 10,000 replicates. Phylogenetic trees were done with the freely available Bosque Phylogenetical Analysis Software version 1.7.152, https://bosque.udec.cl.

We used culture-dependent and culture-independent methods to investigate the habitability of the wet clay-rich soils identified at Yungay. Direct DNA extraction and the subsequent massive parallel sequencing of 16S ribosomal RNA gene amplicons showed that the majority of the sequences obtained are phylogenetically close to 30 bacterial and archaeal species (Fig. 5a), thus unveiling a microbial diversity higher than that reported for soils of this area^30,40,55^. We found that the highest diversity of species was among the Actinobacteria (i.e., *Kocuria*, *Arthrobacter* and other uncultured species of unknown affiliation), followed by halophilic archaea (*Halostella*, *Natromonas, Halorussus, Natrinema*), beta- (*Undibacterium, Aquabacterium, Massilia and Ralstonia*) and gamma-proteobacteria (*Acinetobacter johnsonii, Halomonas gudaonensis, Marinimicrobium locisalis*), with a few species of Firmicutes (*Anaeroccocus, Lactobacillus, Tepidibacter, Streptococcus*), cyanobacteria (*Halothece*) and Bacteroidetes (*Salinibacter*). Some of these species have been already reported in Yungay soils and other regions of the Atacama, such as *Halothece*^56^, *Corynebacterium*^57,58^, *Kocuria*^59^, *Lactobacillus* and *Streptococcus*^60,61^, *Anaerococcus*^62^, *Halomonas* and *Salinibacter*^38,58,62^. Interestingly, we recently reported *Halomonas gudaonensis, Marinimicrobium locisalis* and *Acinetobacter johnsonii* as three of only four microbial species able to reproduce in the extremely rare temporary lagoons that formed after the very unusual rains in Yungay in 2017^46^. This suggests that both ecosystems (subsurface clay soils and surface lagoons) were temporarily connected (temporarily, as these lagoons had completely evaporated at the time of our latest pits dug on 2018), thus suggesting that these three microbial species may have originated from the subsurface wet clay soils reported here. The analyses of microbial species in these lagoons showed that high rainfall events annihilated most surface species in Yungay^46^, however, the diversity of microorganisms in the wet clay-rich soils suggests that the subsurface microbial community in Yungay thrives or at least remained unaffected following high rainfall events. Cultivation of clay-rich samples in different growing media allowed the growth of 23 novel halotolerant bacterial isolates (Fig. 5b), with many species phylogenetically close to genera not detected by direct DNA extraction, such as *Oceanobacillus, Lysinibacilus, Virgibacillus, Halobacillus* and *Bacillus*. Similar to the species detected by direct DNA extraction from clay-rich samples, the microbial species found by cultivation also match the halotolerant/halophilic species reported by other authors in Yungay, like *Halobacillus*^63^; and also other surface and subsurface soils in the Atacama (*Halobacillus*, *Virgibacillus, Oceanobacillus,* and *Bacillus*^64^. The case of *Bacillus* spp. is worth mentioning, as species of this genus are among the first and one of the most common type of bacteria reported in Yungay^27,65,66^ and also in other sites of the hyperarid core of the Atacama^67–69^.

**Figure 6:**
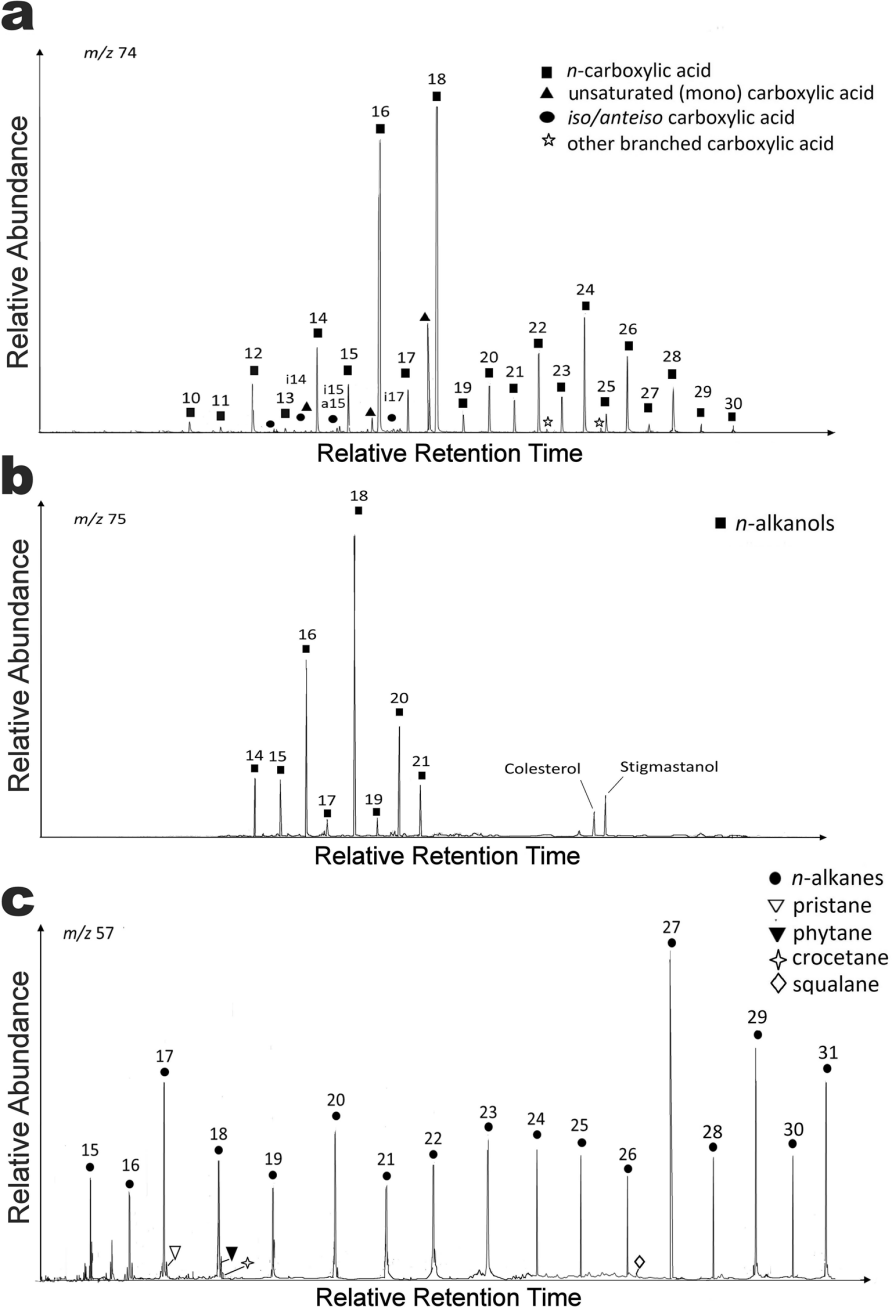
Mass chromatograms of the three major lipid families in Yungay subsurface clay samples. (**a**) Carboxylic acids as methyl esters (m/z 74). (**b**) *n*-alkanols as trimethyl-silyl esters (m/z 75). (**c**) *n*-alkanes (m/z 57).

With the aim of helping inform decisions for the upcoming inspection of Martian smectite by NASA and ESA rovers, we also analyzed what type of biosignatures could be detected in the inhabited wet subsurface smectites of Yungay (Fig. 6a–c). The lipid analysis from clay-rich samples confirmed the presence of microbial biosignatures (heptadecane and phytol derivates) typically associated with different microorganisms such as sulfate-reducing bacteria (*iso*/*anteiso* carboxylic acids), archaea (squalene and crocetane), green non-sulphur bacteria and interestingly, also, cyanobacteria (Fig. 6a,c). Thus, these results not only unveiled the biosignatures produced by the microbial species that inhabit the subsurface clay minerals reported here, but also the molecular remains produced by the microbial species reported in the halites present on the surface^40,70,71^. This is the case of *Halothece* (also detected through Next Generation Sequencing; NGS), of particular interest because it is the only cyanobacteria reported inside halites on the surface soils of Yungay^71^ (the presence of cyanobacteria in the inspected clay-rich samples is ruled out as light does not penetrate 40 cm below the surface, see Supplemental Table S1). The detection of biosignatures of this cyanobacterium on the clay layer lends additional support to the hypothesis that primary carbon fixation in this community can be at least partially explained by the downward seepage of Calvin cycle products synthetized at the surface.

*Iso*/*anteiso* pairs of the C_15_ and C_17_ *n*-carboxylic acids were relatively abundant in the acidic fraction of the analyzed clay-rich samples (Fig. 6a), consistent with the species reported by NGS sequencing. These are lipidic components characteristic of sulphate-reducing bacteria membranes^72,73^ also found in heterotrophic bacteria such as *Thermus*, *Deinococcus* or *Bacillus*^74^. The detection of monounsaturated carboxylic acids such as 16:1ω7; 16:1ω9 or 18:1ω9 are also related to the presence of phototrophic bacteria^74–76^. The dominance of the C17 chain within the low molecular-weight *n*-alkanes (Fig. 6c) and the presence of branched (mostly mono-methylated) *n*-alkanes supported the presence of cyanobacterial sources too^77–80^, as previously described in other surface sites of the Atacama^46,80^. The detection of pristane and phytane also supports the input from surface cyanobacteria, as both isoprenoids are widely viewed as transformation products of phytol^81^. However, other than cyanobacterial origin, pristane can also be sourced in plants or phytoplankton^82^, and phytane can be sourced in archaeol, the most commonly reported core lipid in archaea^31,83^. The contribution from archaea may also be inferred from the presence of squalene and crocetane (although squalene can also be synthetized by fungi), other isoprenoids characteristically attributed to halophilic^84,85^ or methanogenic/methanotrophic^85^ archaea, in line with the archaeal species found by NGS sequencing. In addition to the dominant microbial biomarkers, a contribution from eukaryotes was also inferred from the detection of certain terrestrial lipid biomarkers such as stigmastanol^86,87^ (Fig. 6b) and odd high molecular-weight *n*-alkanes (i.e. C27, C29, and C31^88,89^ (Fig. 6a,c). Because these are highly recalcitrant biomolecules, able to resist decay even for billions of years^83^, and considering that the samples were collected at 40 cm depth, their presence may be potentially explained by the aerial input of allochthonous material coming from elsewhere in the distant past^30,69,80^. The aerial transport of such recalcitrant biomolecules have been described in the Atacama in places such as the neighboring Salar Grande^80^ and in other polyextreme hydrothermal environments such as Dallol in Ethiopia^85^. Altogether, the lipid analysis revealed presence of microbial biomarkers produced by the microorganisms inhabiting the subsurface illite–smectite and those inhabiting surface halites^40,56,71^. The use of source-specific lipid biosignatures in these samples allowed us to elucidate the structure of the microbial community and the different carbon assimilation pathways operating in the clay-rich microenvironment. As already mentioned, the biomass isotopic ratio of δ^13^C measured in the biomass (i.e., TOC) of the clay-rich samples was − 23.4‰. Assuming a mean δ^13^C of − 8‰ for the fixed atmospheric CO_2_^90,91^, the observed δ^13^C value corresponded to a fractionation of *ca*. 15‰, which is within the range (δ^13^C from − 11 to − 26‰) of those described for microorganisms assimilating CO_2_ by the Calvin cycle^92^. A depletion of δ^13^C at C_17_ (− 28.4 ‰) compared to that at C_16_ (− 26.5 ‰) or C_18_ (− 27.0 ‰) suggests a different source for C_17_ homologue relative to the other two low molecular-weight *n*-alkanes. The dominance of C_17_ among the low molecular-weight *n*-alkanes (Fig. 6) is likely related to an input source of cyanobacteria^81,93,94^. δ^13^C compound specific isotope analysis for higher *n*-alkanes (C_27_ and C_29_) rather reflects a possible eukaryotic input^94^. Thus, the analysis of Yungay clay-rich soils suggests that the distribution of *n*-alkanes and other biosignatures also reflect a mixed contribution from different types of microorganisms including cyanobacteria that inhabited surface halite and exogenous eukaryotic material. These results highlight the ability of smectite to preserve different types/sources of biosignatures in an environment as extreme as the hyperarid core of the Atacama, with direct implications for the preservation and discovery of biosignatures on Mars^95^.

Finally, we compared the analytical capabilities of the Gas Chromatography-Mass Spectrometry (GC–MS) techniques used on the Yungay clay minerals with the analytical instruments on the Mars rovers *Curiosity*, *Rosalind Franklin*, and *Perseverance*. The Sample Analysis at Mars (SAM) instrument suite on *Curiosity* can detect a wide range of biosignatures, including volatile organics, polar and non-polar organics, complex organics (> 20 C atoms/molecule) and refractory organics^96^. SAM is a suite of three instruments that analyze volatiles released from rock or soil samples using three experimental methods: (1) evolved gas analysis-quadrupole mass spectrometry (EGA-QMS), in which samples are heated to ~ 800 °C and evolved gases are measured by QMS and GC–MS; (2) combustion, in which samples are heated in the presence of O_2_ and the products are measured by QMS, GC–MS and/or tunable laser spectrometry (TLS); and (3) wet chemistry experiments, in which samples are derivatized at lower temperatures (up to ~ 300 °C)^24,96^. Onboard the *Rosalind Franklin* rover, biomarkers may be detected with a mass spectrometer, the Mars Organic Molecule Analyzer (MOMA), and a Raman spectrometer. MOMA is able to operate in two modes: pyrolysis/gas chromatography mass spectrometry (pyr/GC–MS) or laser desorption/ionization mass spectrometry (LDI-MS) at ambient Mars pressures^97,98^. The Mars2020 *Perseverance* rover, on the other hand, will carry a UV Raman spectrometer, the Scanning Habitable Environments with Raman and Luminescence for Organics and Chemicals (SHERLOC), using fine-scale imaging and a UV laser for the search of biomarkers^99^. Lipid biomarkers (alkanes, isoprenoids, fatty acids or alcohol series) detected with GC–MS techniques in Yungay clay-rich samples and relevant to the search for (a priori prokaryotic) Martian life, are in principle identifiable with the SAM, MOMA and SHERLOC instruments. In fact, lipid detection by SHERLOC should be used as a criterion for sample collection by *Perseverance*. Of the four fundamental biomolecules (lipids, nucleic acids, proteins, and carbohydrates), lipids have the greatest preservation potential over geologic time^100^. Carbonaceous chondrites could have carried abiogenic organic compounds to the Martian surface^101^, which can complicate the detection of biomarkers on Mars. The detection of an even-over-odd preference for fatty acid methyl esters (FAMEs, the building blocks of lipids), however, would be a strong indicator of biological processes^101,102^, assuming Martian biochemistry functions the same as terrestrial biochemistry. Still, key questions arise concerning relevant aspects such as matrix effects or limits of detection. For the GC–MS technique used in this study, samples require specific pre-treatment after organic solvent extraction and prior to analysis, such as separation of the total lipid extract into different polarity fractions (e.g., acidic, apolar and polar) or derivatization with trans-esterification (e.g., BF3) and tri-methylsilylation (e.g., BSTFA) reagents to convert the acidic and alcohol moieties into GC–MS amenable (i.e., volatile enough) compounds (FAMEs and trimethylsilyl ethers, respectively). SAM can perform wet chemistry experiments with tetramethylammonium hydroxide (TMAH) to make lipids sufficiently volatile to detect with GC-MS^103^ and with N-methyl-N(tert-butyldimethylsilyl) trifluoroacetamide (MTBSTFA) to detect amino and carboxylic acids^96^. In fact, the first wet chemistry experiment was recently performed on active eolian Martian sand and results are being interpreted^104^. *Curiosity* is currently in the smectite-rich Glen Torridon unit, and SAM will perform wet chemistry experiments on a smectite-rich sample to search for complex organics^104^. In contrast, analyses with the MOMA and SHERLOC instruments will not involve such a complex pre-treatment. MOMA has the possibility of a derivatization step prior to GC–MS analysis, however, the lack of a previous fractionation step would affect the sample as a whole, not as polarity-specific moieties. As a spectroscopy technique, SHERLOC does not require any derivatization; however, based on fluorescence criteria, it is a technique susceptible to matrix effects that could complicate the detection of organics. Instrument sensitivity is also a key criterion for biomarker detection. SAM has a detection limit similar to the GC–MS technique used to analyze Yungay clay-rich samples (1 ppb or ng g^−1^). The detection limits for both the MOMA (1 nmol, which could vary between 100–600 ng depending on the molecular weight of the analyzed molecule)^97,98^ and SHERLOC (in the range of ppm) instruments^99^, however, are orders of magnitude greater than SAM. Thus, SAM wet chemistry experiments in the smectite-rich Glen Torridon unit may be our best opportunity to identify biomarkers on Mars prior to sample return. The amount, concentration and distribution of target biomarkers (ppb or ppm) are critical for the success of the two near-future life-detection Martian missions.

A final point to consider for the detection of ancient Martian biomarkers is the alteration of organic molecules over time. The alteration and degradation of organic matter on Earth from burial diagenesis and thermal processes as they relate to hydrocarbon production have been well-studied for decades. Organic matter decomposes during early burial diagenesis typically via oxidation to CO_2_ by microorganisms^105^. With progressive burial, heat and metamorphism, organic matter is first carbonized, removing most noncarbon elements and forming an aromatic skeleton, then graphitized through polymerization and structural rearrangement of the aromatic skeleton^106^. The oldest known sedimentary sequences on Earth (> 3.7 Gyr old) have been metamorphosed such that organic carbon has been graphitized, and whether the graphitized carbon represents traces of early life has been debated^107,108^. Mars, unlike Earth, never had robust plate tectonics^109^ and, as a result, 3–4 Gyr old sedimentary rocks on the Martian surface have, for the most part, been minimally altered since their deposition. Even the ancient smectite-bearing mudstone in Gale crater lacks substantial mineralogical evidence for widespread burial diagenesis (i.e., there is no evidence for illitization)^110^ despite a potential burial depth of up to 5 km^111^. Therefore, ancient smectite-bearing martian surfaces are ideal locations to investigate habitability in our early Solar System. Ancient sedimentary rocks on Mars have experienced some degree of diagenesis because they are lithified, cemented and contain mineralized veins and concretions^112–114^. Diagenesis, in some cases, might work in favor of preserving organic matter on Mars. Thiophenic, aromatic and aliphatic compounds discovered by SAM in Gale crater have sulfur in their structures, suggesting early diagenesis in the presence of reduced sulfur made these molecules recalcitrant^25^. If sulfurization was a widespread process on early Mars, ancient organic molecules may have been preserved across the planet and may be indeed present in Jezero crater and Oxia Planum. Cosmic radiation on the Martian surface would also have a significant effect on biomarkers, especially those in the top 1 m of the surface^115^. Radiation oxidizes hydrocarbons and aromatic macromolecules to organic salts and CO_2_. The detection of organic molecules by SAM in multiple locations in Gale crater and the presence of organic molecules in carbonaceous chondrites, however, indicates that organic matter can survive long-term exposure to ionizing radiation^115^.

## Conclusions

The results presented here, showing that wet subsurface clay minerals are inhabited by a number of metabolically active microorganisms in the midst of the driest place on earth, isolated and protected just centimeters bellow the extremely harsh surface environmental conditions typical of the Atacama, reinforce the notion that early Mars could have been a planet with similar subsurface protected habitable niches, particularly during the first billion years of its history. Orbital and *in-situ* measurements demonstrate a heterogeneous distribution of smectite and other clay minerals in Noachian- and early Hesperian-aged (~ 3.5 Gyr ago) surfaces^116,117^. Computer simulations and geochemical models suggest that clay minerals could have formed during relatively short warmer and wetter cycles in a predominantly dry and/or cold early Mars^118,119^. Thus, much of early Mars’ history may have been similar to the modern Atacama, where wetting events were very rare. Because of colder conditions on early Mars, liquid water may have been generated by melting ice, rather than rain.

The known patchy distribution of subsurface habitats in the hyperarid core of the Atacama^29,120,121^ suggests that the search for biosignatures on Mars with the scientific payloads of future rovers will be a difficult task, as potential biosignatures could be similarly unevenly distributed throughout the Martian subsurface. Because the subsurface microbial community discovered in Yungay has adapted to an environment of persistent superficial drought with rare exposure to water, this expands potentially habitable environments on Mars to those with evidence for sporadic exposure to water, rather than persistent exposure. Fracture-associated halos in Gale crater, for example, extend into clay-rich rocks and represent locations where multiple fluid episodes altered the rock^122,123^. These clay-rich, subsurface zones may have been habitable to microorganisms similar to those found in subsurface soils in Yungay. If similar zones of aqueous alteration along fractures are discovered in Jezero crater, we suggest these should be high-priority targets for sample selection with *Perseverance* for eventual return to Earth. Our results also have implications for planetary protection constraints^124^, as ExoMars has a 2 m drill that could easily reach undetected, potentially inhabited clay minerals below the Martian surface. Ancient, smectite-bearing rocks, however, may not be as habitable as unlithified soils because there may not be sufficient pore space for microorganisms to inhabit and the structural water in smectite-bearing rock may not be as readily available to microorganisms. The findings reported here may be considered as a guide to the search for hotspots preserving molecular biomarkers, and potentially life, well-protected below the Martian surface.

## Materials and methods

### Sampling sites and dates

Twenty soil samples were first taken in the field on 13 March 2017 using sterile gloves and sterile falcon tubes (Thermo Fisher Scientific, Massachusetts, USA), on a pit dug in the region of Yungay with coordinates 24°5′4.52″S, 69°54′55.11″W. Twenty additional samples were taken from this pit on 8 March 2018. Four other pits were dug on 20 August 2018, with coordinates 24°4′57.9″S, 69°54′55.50″W; 24°5′24.70″S, 69°54′40.50″W; 24°4′37.9″S, 69°54′48.50″W and 24°4′19.46″S, 69°53′58.86″W. Pits were dug to a depth of ~ 70 cm. All samples were stored inside a plastic cooler at room temperature at all times from the sampling site to the lab.

### Light penetration in the soil

Light penetration in the soil was measured in the field with a digital lux meter (Bestone Industrial Co., Ltd., Shenzhen, China). Measurements were taken by leaving the digital lux meter at the soil surface for 1 min, and then burying the sensor portion of the digital lux meter at one, and three centimeters of depth for the same amount of time.

### Environmental characterization (temperature and relative humidity)

Temperature and relative humidity were measured in the field using dual iButton temperature/Humidity micro loggers (Maxim Integrated, San Jose, CA, USA) as previously done^46^, set to take data every 10 min during 28 July and 19 August 2018, and every 5 min between 22 and 29 July 2019. These sensors were set at the soil surface (shaded under a small rock) and also completely buried at 20 and 40 cm depth, with the sensor set at 40 cm well-immersed in the wet clay-rich soil. Environmental data statistical analyses: Pearson correlation coefficient. The number of degrees of freedom for r is n-2, where n is the number of pairs of bivariate data, for this case 782 for each correlation. Level of significance: alpha = 0.05.

### X-ray diffraction

Bulk samples were stored at room temperature and then ground in the lab into powder with an agate mortar and pestle (Pulverisette 2, Fristsch, Idar-Oberstein, Germany), and X-ray powder diffraction data were collected using a Bruker D8 Eco Advance (Massachusetts, USA) in Bragg–Brentano geometry, Cu Kα radiation and Lynxeye XE-T linear detector. The X-ray generator was operated at 40 kV and 25 mA. Samples were scanned with a 0.05° (2θ) step size, over the range 5–60° (2θ) with a 1 s collection time at each step. Phase identification was performed by comparing the measured diffraction pattern (diffractograms) with patterns of the PDF Database with the DIFFRAC.EVA software (Bruker AXS, Massachusetts, USA). Oriented clay mounts were prepared using the filter-peel method^125^.

### Clay characterization

Bulk samples in the lab were stored at room temperature and then dispersed in sodium hexametaphosphate (HMP 5%wt). Size fractionation was performed by low-speed centrifugation to obtain a suspension of the < 2 μm size fraction according to Stokes’ law (also used for clay percentage determination). The suspension of clay-sized particles was titrated (acetate buffer, pH 5.0, until a pH of 6.8 was obtained) and washed with distilled water to remove carbonates and soluble salts, respectively.

### Near-infrared spectroscopy

Near-infrared spectra of the clay-sized separates were stored at room temperature and then analyzed in the lab using the diffuse reflection method^126^ (DRIFT) with a Nicolet FTIR spectrometer (Thermo Fisher Scientific, Massachusetts, USA). Samples were poured into a sample cup without dilution in KBr. Analysis were done at room temperature and under air-dried atmosphere. We used a mirror for background measurement. Spectra were collected using a DTGS-KBr detector at 2 cm^−1^ resolution in the NIR region (from 12,000 to 4000 cm^−1^; 1 to 2.5 μm) with a quartz beamsplitter.

### Bacterial counts

Microorganisms in clay bearing soils were stored at room temperature and then enumerated by direct counts in the bulk samples in the lab. As cell visualization is less efficient when minerals mask cells by adsorbing traditional dyes, we used SYBR Green I (Molecular Probes, Eugene, USA) instead of DAPI (4′,6-Diamidino-2-Phenylindole, Dihydrochloride). Briefly, 2 g of clay samples (incubated for 2 weeks at 37 °C) were suspended in 10 mL of 0.01 M tetrasodium pyrophosphate and gently sonicated in a Branson ultrasonic cleaner (Danbury, USA) for 30 min in ice cooled water. After 10 s of sedimentation, 2 mL of the supernatant were taken and placed directly on black Isopore polycarbonate membrane filter (0.2 μm pore size, Millipore, Massachusetts, USA). Membranes were then incubated with 1.5 mL of SYBR Green I (50 μg/L) during 15 min in the dark. Membranes were then washed with 10 mL of distilled water and left in dark for air drying. One drop of immersion oil was deposited on glass slide, then the filter and another drop of oil was deposited on the filter surface and covered with a glass cover. Bacteria were counted immediately using Zeiss AxioImager M.2 fluorescence microscope (Carl Zeiss, Jena, Germany) and a Plan-Apo 63x ⁄ 1.4 Zeiss oil-immersion objective. Filter set for eGFP (Zeiss Filter Set 38; Excitation ⁄ Emission: 450–490 ⁄ 500–550 nm) was used for SBI stained bacteria visualization. Bacteria were counted by selecting random fields of view, with 20 fields per filter analyzed. Five filters were counted per sample, for a total of 10 filters and 200 fields observed.

### DNA extraction from isolates

Clay samples were stored at room temperature and DNA extracted from them in the lab using the DNeasy UltraClean Microbial Kit (Quiagen, Düsseldorf, Germany) according to the manufacturer instruction and as previously performed, except that at the cell lysis step, two pulses of 2 min were used in a FastPrep-24 5G homogenizer (MP Biomedicals, Irvine, USA).

### Illumina NGS-based 16S rRNA Sequencing

Illumina sequencing in the lab was performed using the DNA extracted from the clay samples by the Fundación Parque Científico Madrid (FPCM, Madrid, Spain) sequencing service using their standard procedures as follows. Bacterial 16S rRNA V3-V4 hypervariable gene region was amplified with the primer pair 341f. (5′CCTACGGGNGGCWGCAG3′) and 805r (5′GACTACHVGGGTATCTAATCC′)^127^. After DNA extraction from soil samples, PCR and Illumina MiSeq (Berlin, Germany), sequencing were carried out at the Genomic Service of FPCM.

Purified DNAs were amplified in a first PCR of 30 cycles with Q5 Hot Start High-Fidelity DNA Polymerase (New England Biolabs, Massachusetts, USA) in the presence of 100 nM primers S-D-Bact-0341-b-S-17 (5′-ACACTGACGACATGGTTCTACACCTACGGGNGGCWGCAG-3′ and S-D-Bact-0785-a-A-21 5′-TACGGTAGCAGAGACTTGGTCTGACTACHVGGGTATCTAATCC-3′), for the amplification of the V3-V4 region of 16S rRNA. After the first PCR, a second PCR of 15 cycles was performed with Q5 Hot Start High-Fidelity DNA Polymerase (New England Biolabs, Massachusetts, USA) in the presence of 400 nM of primers 4242A1 (5′-AATGATACGGCGACCACCGAGATCTACACTGACGACATGGTTCTACA-3′ and 806R 5′-CAAGCAGAAGACGGCATACGAGAT-(10 nucleotides barcode)-534R TACGGTAGCAGAGACTTGGTCT-3′) of the Access Array Barcode Library for Illumina Sequencers (Fluidigm, San Francisco, USA). The finally obtained amplicons were validated and quantified by Bioanalyzer and an equimolecular pool was purified using AMPure beads (Beckman Coulter, Pasadena, USA) and titrated by quantitative PCR using the Kapa-SYBR FAST qPCR kit for LightCycler480 (Kapa Biosystems, Cape Town, South Africa) and a reference standard for quantification. The pool of amplicons was denatured prior to be seeded on a flowcell at a density of 10 pM, where clusters were formed and sequenced using a MiSeq Reagent Nano Kit v2 (Illumina, Berlin, Germany) in a 2 × 250 pair-end sequencing run on a MiSeq sequencer (Illumina, Berlin, Germany).

Raw sequences were processed in MOTHUR software v.1.40.5^128^, using a custom script based on MiSeq SOP^129^. Briefly, reads below 400 bp, with ambiguous nucleotide identities and/or homopolymers longer than 8 bp, singletons and putative chimeras were removed from subsequent analyses. Remaining reads were then clustered into OTUs (Operational Taxonomic Units) at the 97% similarity level. Number of sequences were finally normalized among samples to the lesser number of reads, i.e. 9823, by random selection. Taxonomic assignations were performed by comparing OTU’s representative sequences with RDP database^130^, as it is the most up-to-date database that can be included in MOTHUR analyses, as well as the most accurate in assigning real taxonomic affiliation of OTUs obtained from desert samples^79^. OTUs assigned to ‘cyanobacteria/chloroplast’ were further compared with NBCI GenBank, EMBL, Greengenes and SILVA databases for more precise cyanobacteria taxonomic identification.

### Cultivation of isolates from clay samples

In the lab, clay samples were stored at room temperature and then aseptically inoculated in Petri dishes containing agar and either Luria–Bertani Broth (Sigma-Aldrich, Missouri, USA) or Marine Media (CondaLab, Torrejón de Ardoz, Spain). Colonies arising from clay particles usually were evident 2–3 days after inoculation. These colonies were then re-cultivated in the media from they were first isolated to obtain enough biomass for DNA extraction and storage. *Lysinibacillus* and *Oceanobacillus* species were able to grow both in Luria–Bertani Broth and Marine Media agar plates, while all other species were able to grow only in Luria–Bertani Broth agar plates. The a_w_ measured inside sealed Petri dishes containing Luria–Bertani Broth agar was 0.985, while the a_w_ measured inside sealed Petri dishes containing Marine Media agar plates was 0.973.

### 16S rRNA amplification and sequencing from isolates DNA

As previously performed^69^, 16S rRNA of bacterial isolates was amplified in the lab using the GoTaq Green Master Mix (Promega, Wisconsin, USA) and the primers 341f. (5′CCTACGGGNGGCWGCAG3′) and 785r (5′GACTACHVGGGTATCTAATCC′). PCR conditions used were: 95 °C for 5 min, and 25 cycles of (95 °C for 40 s, 55C for 2 min, 72 °C for 1 min) followed by 72 °C for 7 min. The resultant reactions were visualized in a 2% agarose TAE gel at 50 V.

The automated sequencing of the resulting PCR products was conducted by Macrogen DNA Sequencing Inc. (Seoul, Korea). Sequences were checked for quality using the BioEdit software (Ibis Therapeutics, Carlsbad, USA) and end-trimmed before using the Megablast option for highly similar sequences of the BLASTN algorithm against the National Centre for Biotechnology Information nonredundant database (www.ncbi.nlm.nih.gov) to search for the closest species of each of the isolates obtained. Only species with at least 98% of sequence identify and an E value of 0.0 were selected, and only species with defined genus and species names were considered for phylogenetic closeness.

### Phylogenetic analysis

In the lab, phylogenetic analysis of 16S rRNA gene sequences obtained from isolates were aligned by multiple sequence comparison by log-expectation^131^, analyzed with jModelTest^132^ and then by Phylip NJ (bootstrap 10,000)^133^, all tools of the freely available Bosque phylogenetic analysis software (version 1.7.152)^134^.

### Fluorescence microscopy, SYBR green and CTC staining

Small fragments of moist clay aggregates from Yungay (about 1 cm^3^) were carefully cut with a sterile blade in the lab. Plane clay surfaces were stained during 30 min at 37 °C in dark with 100 μL (1:100 dilution) of SYBR Green I (SBI) (Molecular Probes, Eugene, USA), a fluorochrome specific for staining nucleic acids. Multichannel Image Acquisition (MIA) system was used with combination of following filter sets: filter set for eGFP (Zeiss Filter Set 38; Ex ⁄ Em: 450–490 ⁄ 500–550 nm) and Rhodamine (Zeiss Filter Set 20; Ex ⁄ Em: 540–552 ⁄ 567–647 nm) and autofluorescence signal potentially proceeded from extracellular polymeric substances (EPS), respectively. The eGFP and DAPI (Zeiss Filter Set 49; Ex ⁄Em: 365 ⁄ 420–470 nm) filter sets were used for acquisition of single section images of SBI stained nucleic acids and autofluorescence signal potentially proceeded from EPS, respectively. Likewise, plane clay surfaces were stained during 24 h at 25 °C in dark with 100 μL of 5 mM of tetrazolium salt: 5-cyano-2,3-ditolyl tetrazolium chloride (CTC) (Molecular Probes, Eugene, USA)^135^. The formazan crystals are viewed as yellow-orange fluorescent spots when using Ex ⁄ Em: 426–446 ⁄ 545–645 nm filter set.

After both stains, a drop of immersion oil was placed on plane clay surfaces. After covering a clay surface with a square coverslip the samples were visualized with Zeiss AxioImager M.2 fluorescence microscope (Carl Zeiss, Jena, Germany) and a Plan-Apo 60x ⁄ 1.4 Zeiss oil-immersion objective. In some cases for better 3D visualization of bacteria aggregates, the Maximum Image Projection (MIP) images were reconstructed using the software package AxioVision 4.8.1 (Carl Zeiss, Jena, Germany).

EPS are a complex mixture of biomolecules; polysaccharides, proteins, nucleic acids, lipids and other macromolecules. Proteins and exopolysaccharides represent the key components of EPS, accounting for 40–95% of its structure. There are at least two components of EPS which can produce a signal in fluorescence microscopy; proteins and nucleic acids. We used SYBR Green staining for staining bacterial nucleic acids, thus disperse nucleic acids in the EPS should also be stained, leading to a detectable, although weaker, signal in FM. Some clays can produce a very weak autofluorescence signal, however, we did not observe any clay autofluorescence, but only the autofluorescence of proteins and the fluorescence coming from the SYBR Green stained nucleic acids.

### Biosignature analyses

Biosignatures analysis were performed as previously reported by us^136^.

A. Geolipid extraction, fractionation and analysis
  Sediment sample (~ 50 g) was extracted with a mixture of dichloromethane/methanol (DCM/MeOH, 3:1, v/v) for 24 h with a Soxhlet apparatus (Fisher Scientific, New Hampshire, USA). Internal standards (tetracosane-D50, myristic acid-D27, 2-hexadecanol) were added prior to extraction. The total lipid extracts were concentrated by rotary evaporation to 2 ml. After this step, activated copper was added and allowed to stand overnight to remove elemental sulfur. The extracted sample was separated in two fractions using a Bond-elute column chromatography (bond phase NH2, 500 mg, 40 µm particle size). The neutral lipid fraction was obtained by eluting with 15 ml DCM/2-propanol (2:1,v/v) and the acid fraction with 15 ml of acetic acid (2%) in diethyl ether. Further separation of the neutral lipid fraction was done with 0.5 g of alumina in a Pasteur pipet. The non-polar fraction was obtained by eluting 4.5 ml of hexane/DCM (9:1, v/v) and the polar fraction with 3 ml of DCM/methanol (1:1, v/v). The acid fraction was derivatized with BF3 in methanol and the polar fraction with N,O-bis (trimethylsilyl) trifuoroacetamide (BSTFA).
B. Gas chromatography-mass spectrometry (GC–MS) analysis
  The sample (non-polar, acid, and polar fraction) was analyzed by gas chromatography mass spectrometry using a 6850 GC system coupled to a 5975 VL MSD with a triple axis detector (Agilent Technologies, Santa Clara, USA) operating with electron ionization at 70 eV and scanning from 50 to 650 m/z. The analytes were injected (1 μl) and separated on a HP-5MS column (30 m × 0.25 mm i.d. × 0.25 μm film thickness) using He as a carrier gas at 1.1 ml min^−1^. For the non-polar fraction, the oven temperature was programmed from 50 to 130 °C at 20 °C min^−1^ and then to 300 °C at 6 °C min^−1^ (held 20 min). For the acid fraction the oven temperature was programmed from 70 to 130 °C at 20 °C min^−1^ and then to 300 °C at 10 °C min^−1^ (held 10 min). For the polar fraction the oven temperature program was the same as that for the acid fraction, but the oven was held for 15 min at 300 °C. The injector temperature was 290 °C, the transfer line was 300 °C, and the MS source was 240 °C. Compound identification was based on the comparison of mass spectra and/or reference compounds, compounds were quantified with external calibration curves. External standards of *n*-alkanes (C10 to C40), fatty acids methyl esters, FAMEs (C8 to C24), alkanols (C10, C14, C18, C20), and branched isoprenoids (2,6,10-trimethyl-docosane, crocetane, pristane, phytane, squalane and squalene) were injected to obtain calibration curves. Recoveries of the internal standards averaged 69 ± 18%.
C. Stable isotopes analysis: organic carbon and total nitrogen
  Total nitrogen (δ^15^N) and organic carbon (δ^13^C) isotopes were measure by USGS methods^137^. Briefly, sediment sample (2 g) was homogenized by grinding with a corundum mortar and pestle. Then HCl was added to 0.5 g of sample to remove carbonate, equilibrated for 24 h, and adjusted to neutral pH with ultrapure water, dried at oven (50 °C), and analyzed in the IRMS (MAT 253, Thermo Fisher Scientific, Massachusetts, USA). δ^13^C and δ^15^N values were reported in the standard per mil notation using three certified standards (USGS41, IAEA-600, and USGS40), analytical precision of 0.1‰.
D. Compound specific isotope analysis

Conditions for gas chromatography analysis of isotopic ratio of *n*-alkanes, were identical with standard GC–MS analysis for the polar fraction. Carbon isotopic composition of individual compounds were performed using an isotope ratio gas-chromatograph–mass spectrometry (GC-IRMS) system, a Trace GC 1310 ultra, coupled to a MAT 253 IRMS (Thermo Fisher Scientific, Massachusetts, USA). Conditions in the IRMS were as follows: electron ionization 100 eV, Faraday cup collectors m/z 44, 45 and 46, a CuO/NiO combustion interface maintained at 1000 °C. The samples were injected in splitless mode (inlet temperature 250 °C, helium as a carrier gas at constant flow of 1.1 ml min^−1^). The isotopic values of peaks produced in the combustion reactor of the chromatography separated compounds were calculated using CO_2_-spikes of known isotopic composition, introduced directly into the source three time at the beginning and at the end of every run. An *n*-alkane reference mixture with known isotopic composition (A6, Indiana University, USA) were run after every four samples to check accuracy of the isotopic ratio determined by the GC-IRMS.

## Supplementary information

Supplementary Information

## Data Availability

Data and materials are available, and should be requested to A.A-B.
