# Supplementary material for: Inhabited subsurface wet smectites in the hyperarid core of the Atacama Desert as an analog for the search for life on Mars
Source: Sci Rep. 2020 Nov 5;10:19183. doi: 10.1038/s41598-020-76302-z (PMC7645800; doi:10.1038/s41598-020-76302-z)
Supplement: Supplementary file 1 — Supplementary Information [file 41598_2020_76302_MOESM1_ESM.pdf]

Inhabited subsurface wet smectites in the hyperarid core of the Atacama Desert as an analog for the search for life on Mars

Armando Azua-Bustos[1,2*], Alberto G. Fairén[1,3*], Carlos González Silva[4], Daniel Carrizo[1], Miguel Ángel Fernández-Martínez[1,5], Cristián Arenas-Fajardo[6], Maite Fernández-Sampedro[1], Carolina Gil-Lozano[1,7], Laura Sánchez-García[1], Carmen Ascaso[8], Jacek Wierzchos[8], and Elizabeth B. Rampe[9].

[1]Centro de Astrobiología (CSIC-INTA), 28850 Madrid, Spain.
[2]Instituto de Ciencias Biomédicas, Facultad de Ciencias de la Salud, Universidad Autónoma de Chile, Santiago, Chile.
[3]Department of Astronomy, Cornell University, Ithaca 14853 NY, USA.
[4]Facultad de Ciencias, Universidad de Tarapacá, Chile
[5]Department of Natural Resource Sciences, McGill University, Quebec, Canada.
[6]Atacama Research, Santiago, Chile.
[7]Laboratory of Planetology and Geodynamics, Université de Nantes, 44322 France.
[8]Museo Nacional de Ciencias Naturales (CSIC), 28006 Madrid, Spain.
[9]Astromaterials Research and Exploration Science Division, NASA Johnson Space Center, Houston, TX, USA.

*Corresponding authors: Armando Azua-Bustos (aazua@cab.inta-csic.es) (to whom correspondence should be addressed) and Alberto G. Fairén (agfairen@cab.inta-csic.es) Centro de Astrobiología (CSIC-INTA), Madrid, Spain.

Supplementary Information

Supplemental Tables and Figures

Supplemental Table S1. Light penetration in the soil profile at Yungay.

Surface	1cm	3cm
124000	36	0
125000	30	0
125000	43	0

Light penetration (lux) was measured three times between 12:40 and 13:00.

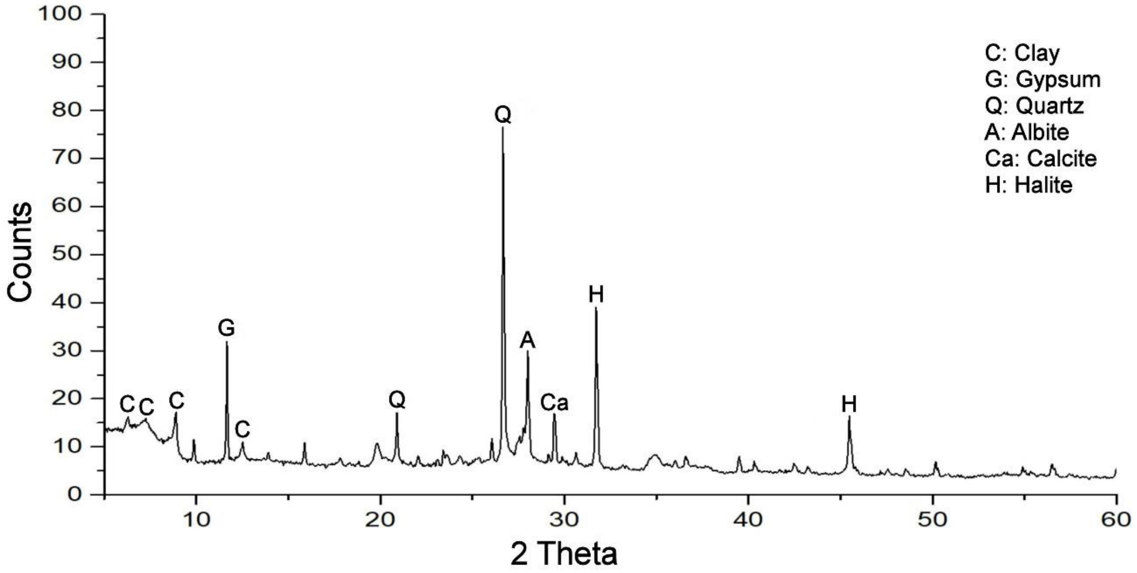

Supplemental Figure S1. Bulk powder XRD pattern of Yungay clay-rich sample collected at 40 cm depth.

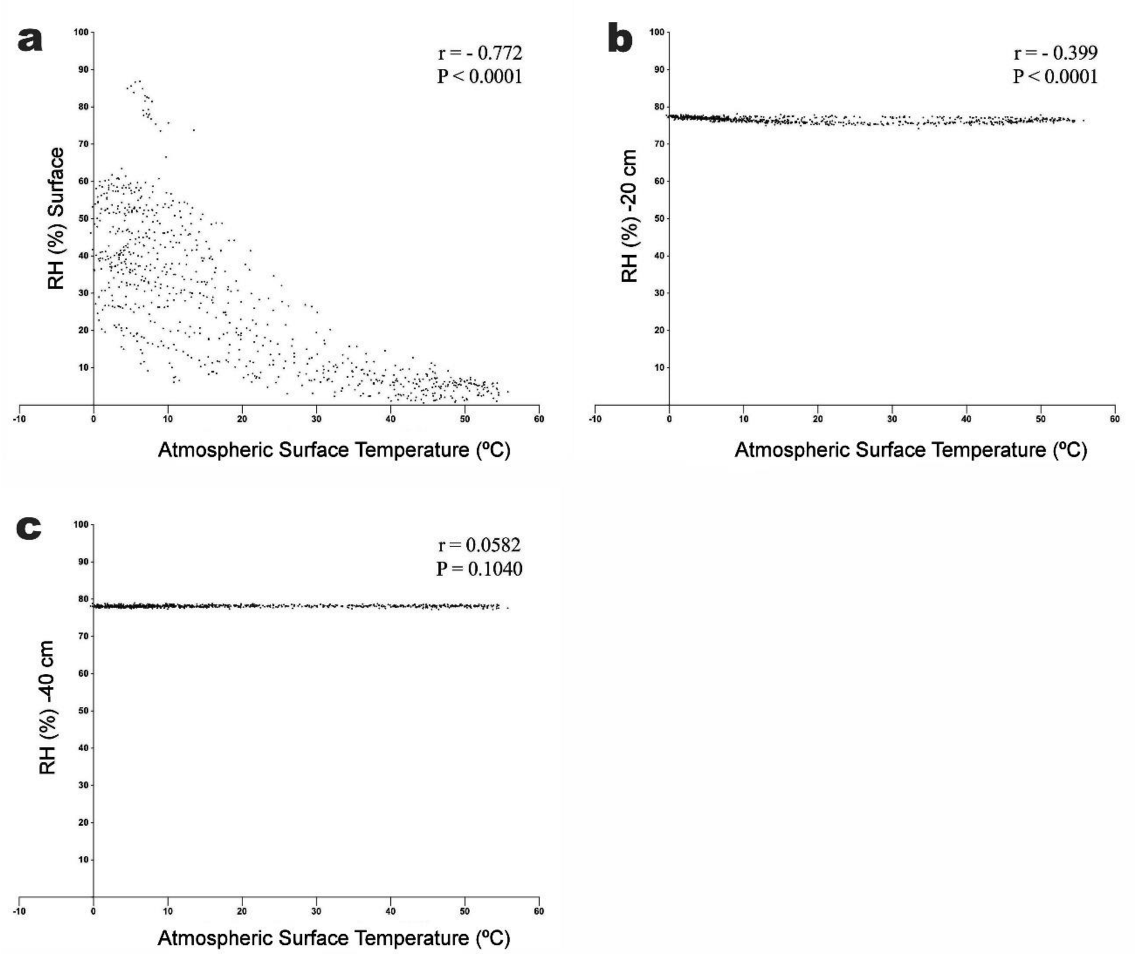

Supplemental Figure S2. Pearson correlation coefficient between atmospheric external temperatures and relative humidity at each soil depth. (a) Atmospheric temperature vs surface relative humidity. (b) Atmospheric temperature vs relative humidity at -20 cm. (c) Atmospheric temperature vs relative humidity at -40 cm.
